# Results of a feasibility randomized controlled trial (RCT) of the Toolkit for Optimal Recovery (TOR): a live video program to prevent chronic pain in at-risk adults with orthopedic injuries

**DOI:** 10.1186/s40814-019-0416-7

**Published:** 2019-02-20

**Authors:** Ana-Maria Vranceanu, Cale Jacobs, Ann Lin, Jonathan Greenberg, Christopher J. Funes, Mitchel B. Harris, Marilyn M. Heng, Eric A. Macklin, David Ring

**Affiliations:** 1 Integrated Brain Health Clinical and Research Program, Department of Psychiatry, Massachusetts General Hospital, One Bowdoin Square, 1st Floor, Boston, MA 02114 USA; 2 Harvard Medical School, Boston, MA USA; 3 Department of Orthopedic Surgery, University of Kentucky Medical Center, Lexington, KY USA; 4 Department of Orthopedic Surgery, Massachusetts General Hospital, Boston, MA USA; 5 Biostatistics Center, Massachusetts General Hospital, Boston, MA USA; 6 Department of Surgery and Perioperative Care, The University of Texas at Austin Dell Medical Center, Austin, TX USA; 7 The University of Texas at Austin Dell Medical School, Austin, TX USA

**Keywords:** Orthopedic musculoskeletal injury, Prevention of chronic pain, Physical function, Intervention, Video, Telehealth

## Abstract

**Background:**

Orthopedic injuries are the leading cause of hospital admissions in the USA, and many of these patients transition into chronic pain. Currently, there are no evidence-based interventions targeting prevention of chronic pain in patients with orthopedic injuries. We iteratively developed a four-session intervention “The Toolkit for Optimal Recovery” (TOR) which we plan to subsequently test for efficacy in a phase III hybrid efficacy-effectiveness multi-site clinical trial. In order to prevent methodological weaknesses in the subsequent trial, we conducted a feasibility pilot to evaluate the TOR delivered via secure live video versus usual care (UC) in patients with orthopedic injuries from an urban, level I trauma clinic, who screen in as at risk for chronic pain and disability. We tested the feasibility of recruitment, acceptability of screening, and randomization methods; acceptability of the intervention, treatment adherence, and treatment fidelity; satisfaction with the intervention; feasibility of the assessment process at all time points; acceptability of outcome measures for the definitive trial; and within-treatment effect sizes.

**Methods:**

We aimed to recruit 50–60 participants, randomize, and retain them for ~ 4 months. Assessments were done electronically via REDCap at baseline, post-intervention (approximately 5 weeks after baseline), and 3 months later. We followed procedures we intend to implement in the full-scale hybrid efficacy-effectiveness trial.

**Results:**

We recruited 54 participants and found that randomization and data collection procedures were generally acceptable. The majority of participants were white, educated, and employed. Warm hand-off referrals were more effective than research assistants directly approaching patients for participation without their providers’ engagement. Feasibility of recruitment, acceptability of screening, and randomization were good. Satisfaction with the program, adherence to treatment sessions, and treatment fidelity were all high. There were no technical issues associated with the live video delivery of the TOR. There was minimal missing data and outcome measures were deemed appropriate. Effect sizes for improvement after participation in TOR were moderate to large. There were many lessons learned for future trials.

**Conclusions:**

This study provided evidence of the feasibility of the planned hybrid efficacy-effectiveness trial design when implemented at our home institution. Establishing feasibility of the intervention and study procedures at other trauma centers with more diverse patient populations and different clinical practices is required before a multi-site phase III efficacy-effectiveness trial.

**Trial registration:**

ClinicalTrials.gov ID: NCT03405610. Registered on January 28, 2018—retrospectively registered.

## Background

Musculoskeletal injuries (e.g., fractures, dislocations, etc.; also known as traumatic injuries) represent the leading cause of adult hospital admissions [1], with many patients developing chronic pain and disability [2] despite adequate recovery of their bones and soft tissues. These patients may continue to have multiple surgeries and medical appointments, resulting in increased health care costs and a significant public health burden [3, 4].

Catastrophic thinking about pain, pain anxiety, and depression are established risk factors for disability and pain in patients with traumatic musculoskeletal injuries [5–11], regardless of the severity of the injury [12, 13]. Recognizing these psychosocial factors early in the recovery process creates a window of opportunity to identify and intervene with patients who are at risk for chronic pain and disability in the acute phase when psychosocial treatments are most effective [14, 15]. A recent systematic review conducted by our team showed that there are no evidence-based interventions that address psychosocial factors in patients recovering from acute orthopedic injuries [16].

Current usual care for patients with acute traumatic musculoskeletal injuries consists primarily of surgical interventions and pain medications. However, medical care is undergoing a shift in priorities recognizing the multifactorial influences on successful recovery after injury and the pivotal role of psychosocial factors [17]. Although surgeons are now aware of the importance of psychosocial factors in recovery after musculoskeletal injuries [18], they are often uncomfortable referring patients for outpatient care [18–20]. Referrals are often done when the pain has already become chronic, patients have undergone multiple medical treatments, and psychological treatments are generally less efficacious [14, 15]. Timely psychological care is also challenging due to long wait times and lack of trained providers. Traditional mental health treatments like cognitive behavioral therapy are met with resistance due to the stigma associated with mental health concerns [10]. Patients with orthopedic injuries are also unable to travel to clinics for medical appointments and have to rely on family and friends for transportation.

To bypass these barriers, using feedback from patients and surgeons, we iteratively developed a four-session live video mind-body program “Toolkit for Optimal Recovery” which we aim to test in a phase III multi-center hybrid efficacy-effectiveness trial [21, 22]. In the current study, we followed recommendations for rigorous feasibility testing [23, 24] and conducted preliminary work needed to inform the study design and identify and rectify potential shortcoming in study procedures and measurement. We sought to determine the best ways to recruit patients with orthopedic traumatic musculoskeletal injuries, estimate loss to follow up, determine acceptability of randomization and assessment procedures, assess adherence to home practice, and determine satisfaction with the program.

## Methods

### Design

The study was designed with the goal of preparing for a future large multi-center hybrid efficacy-effectiveness RCT of the TOR versus UC. In this pilot feasibility study, we conducted the RCT at a level I trauma center at a major urban medical teaching hospital. Randomization was performed using a random number generator to maintain balance between the groups. Participants completed questionnaires electronically with the secure web-based data collection platform REDCap [25]. As comparisons were made with usual care (UC), participants were not blinded to intervention versus control. The trial was designed to address specific objectives relating to study design and methodology for the subsequent hybrid efficacy-effectiveness trial. Consistent with recommendations for rigorous clinical trials, it was not designed to determine efficacy [23, 24]. We collected data at baseline, 4–5 weeks after baseline, and 4 months post-baseline (3 months after post-test). There were no important changes to methods (e.g., eligibility criteria) after the trial commenced.

### Intervention

The TOR is a four-session, live video, manualized mind-body program informed by the fear avoidance model [26, 27]. It combines relaxation response skills (e.g., breath awareness, body scan) with cognitive behavioral skills (e.g., adaptive thinking, activity pacing) and acceptance and commitment therapy skills (e.g., acceptance and value-based goals). Table 1 provides a description of program components. Each session starts with a description of the skills previously learned. Patients are asked to practice skills at home and email a practice log to their clinician prior to the next session. Given known barriers to psychosocial care in this population, great attention is placed on building rapport, normalizing pain after injury, and ensuring that participants’ experiences are validated while instilling hope. Patients learn about the mind-body connection and the importance of practicing mind-body skills to optimize recovery.

**Table 1:** Session content for the “Toolkit for Optimal Recovery” (TOR)

Session	Toolkit material
1	Discuss treatment rationale and goals Review and correct misconceptions about recovery trajectory after orthopedic injury Normalize pain after an injury. Move patients away from the mind-body dichotomy by discussing how all pain sensations originate in the brain. Discuss the difference between “hurt” versus “harm”
	Learn how the sympathetic nervous system influences symptoms; learn about the disability spiral and how it can lead to slower recovery and chronic pain after orthopedic injury. Learn physical, emotional, and cognitive factors that can speed or slow recovery after orthopedic injury
	Provide education about the parasympathetic nervous system and relaxation; demonstrate relaxation strategies (diaphragmatic breathing, body scan)
	Set goals for skills practice: practice relaxation strategies daily
2	Practice diaphragmatic breathing
	Review previous material and homework; problem-solve barriers to practice
	Provide education about the biopsychosocial model and mind-body links
	Conduct mindfulness exercise on pain sensations. Assist patients in identifying what thoughts, feelings, and behaviors are triggered by the pain sensations and normalize this experience. Provide education about mindfulness techniques for observing thoughts-feelings-behaviors non-judgmentally.
	Learn decision tree for unhelpful thoughts: adaptive thinking for thoughts that are not true (e.g., “Pain means that I am getting worse”); acceptance, validation/compassion, and letting go for thoughts previously reframed who keep coming back, or for thoughts that are true but not helpful (It is harder to walk right now).
	Set goals for skills practice: practice diaphragmatic breathing, body scan or mindfulness on pain daily, complete at least one decision tree exercise, complete at least three reframing exercises
3	Practice diaphragmatic breathing
	Review previous material and homework; problem-solve barriers to practice
	Learn problem solving skills; assist patients in identifying a problem related to injury and applying problem solving skills
	Learn acceptance strategies; assist patients in identifying when to use reframing vs. problem solving vs. acceptance
	Provide rational for activity pacing; assist patients in setting activity goals consistent with their values; assist patients in applying acceptance, reframing or problem-solving skills to achieve activity pacing goals
	Set goals for skills practice: practice diaphragmatic breathing, body scan or mindfulness daily, complete at least one decision tree exercise, including options for problem solving, acceptance, and reframing; follow activity pacing protocol
4	Practice diaphragmatic breathing
	Review previous material and homework; problem-solve barriers to practice
	Review all skills; assist patients in identifying which skills are being used, how helpful they are, and how they can be implemented in the future
	Interactive quiz to identify improvements patients have made, skills that are being used, skills the patients would like to continue to work on and a plan for continued coping

The TOR was developed based on prior research [5, 6, 10], the fear avoidance theoretical model [26, 27], feedback from an orthopedic surgeon (DR) and the senior author’s (AMV) extensive clinical and research experience with this population. Briefly, we used elements of the NIH stage model [28] and ORBIT models [29] as conceptual frameworks. The first iteration of the program was called Relaxation Response Cognitive Behavioral Program (RRCB) and was designed to have a 4–6-session flexible format [30]. The program was tested in person in an open pilot. Due to challenges in recruitment, we next modified the delivery to a combination of in-person and telephone visits and conducted a pilot feasibility RCT [30]. We performed qualitative exit interviews over the phone with ten patients from the RRCB group using a semi-structures guide. Data was analyzed using grounded theory and was shared with two of the surgeons who provided referrals. Information from these exit interviews and the two surgeons informed modifications in treatment modality (now video), duration (now 45 min/session), number of sessions (now four), and manual language (to foster patient engagement through language that normalizes challenges associated with recovery and avoids the use of medical jargon which some patients did not like). The Toolkit for Optimal Recovery (TOR) manual was next developed and tested with results reported in this manuscript.

### Usual care (UC) control

Usual care involves meetings with surgeons as needed, referrals to occupational or physical therapy, and pain medications. A strict opioid prescription policy is followed at our institution, and it entails a multi-modal approach to pain medication prescribing utilizing non-opioid drugs such as acetaminophen, NSAIDs, and gabapentin; rational opioid prescribing that emphasize low starting doses and early encouragement of a weaning regimen; and monitoring of the state prescription monitoring database. No more than 1 week worth of opioids is provided at discharge or during follow up. All patients received usual care regardless of randomization.

### Recruitment, consent, and screening

In order to determine whether we could recruit the desired number of participants and ascertain which methods or recruitment are most successful at our level I trauma center, we report the number of participants approached and consented. We aimed to enroll 50–60 participants to achieve 50 study completers. Given approximately 200 participants that meet the study criteria available each year, we had at least an 80% probability of demonstrating that recruitment is feasible if the expected proportion of eligible patients who agree to participate is at least 72%. This sample size is sufficient for answering additional feasibility and acceptability questions and is typically used in randomized controlled feasibility trials [31–33]. The research assistant screened the orthopedic trauma clinic of two surgeons who agreed to participate in the study, to identify patients who underwent an orthopedic injury 1–2 months prior to this clinic visit. The research assistant next approached patients for participation in the patient rooms, as they were waiting for their appointment with the surgeon. Interested participants completed informed consent and were subsequently screened for participation. Inclusion criteria included: (1) an orthopedic injury in the prior 1–2 months, (2) 18 years or older, (3) English fluency and literacy, and (4) score over median split on the Pain Catastrophizing Scale (PCS) or Pain Anxiety Symptom Scale (PASS-20). Exclusion criteria included: (1) major medical comorbidity expected to worsen in the next 6 months; (2) comorbid chronic pain conditions; (3) change in antidepressant medication in the past 3 months; (4) evidence for potential secondary gain such as litigations or worker’s compensation procedures that may interfere with patients’ motivation for treatment; (5) psychoses, bipolar disorder, active untreated substance dependence or other factors that could interfere with informed consent processes and treatment (by self-report); (6) inability or unwillingness to complete questionnaires electronically or participate in a mind-body program delivered via secure synchronous live video; and (7) pregnancy, per our Institutional Review Board (IRB). Recruitment methods sampled included: (1) approaching participants with an orthopedic injury in the prior 1–2 months in the waiting room, with no help from the medical team; (2) introduction of the research assistant by the orthopedic surgeon; and (3) introduction of the study by the orthopedic surgeon to individual patients. Recruitment occurred between January 2016 and May 2018. The study protocols (summary and detailed) are stored with the IRB.

### Assessments

Eligible participants completed demographics and a self-report questionnaire electronically. We explored two methods of assessment at baseline: in the office on an iPad or at home through an email link. One of the surgeons allowed completion of screening and assessments before the medical visit, while the other preferred to interrupt the assessment to conduct the medical visits.

*Demographic questionnaires*: assessed age, gender, race/ethnicity, employment status, marital status, educational level, prior orthopedic injuries, current psychotropic or pain medications, and history of psychological diagnoses.

*The Short Musculoskeletal Function Assessment Questionnaire* (*SMFA*) [34] is a validated 46-item questionnaire that measures physical functioning/musculoskeletal disability [11, 35]. It is developed from the 101-item parent questionnaire which has been extensively validated and tested for reliability and responsiveness [36]. The score is calculated by summing up the individual items which cover assessment of function (34 questions) and perception of how bothersome symptoms are (12 questions). All questions are answered on a 4-point Likert scale with high scores depicting higher disability. Raw scores are summed and transformed so that the final score ranges from 0 to 100. Internal reliability was excellent (Cronbach’s *α* = 0.91). SMFA will be our primary outcome in the definitive efficacy-effectiveness trial.

*The Numerical Rating Scale* (*NRS*) was used to assess pain at rest and with activity. This is a commonly used, validated, and reliable measure of pain intensity [37–40] on an 11-point item scale from “no pain” (0) to “worst pain imaginable” (10). We assessed both pain at rest and with activity.

*The Pain Catastrophizing Scale* (*PCS*) [41, 42] is a reliable and valid measure of negative pain-related cognitions or catastrophic thinking (thinking of the worst). It has 13 items answered on a 4-point Likert scale from “not at all” (0) to “all of the time” (3). Items are grouped into three subscales: rumination (tendency to spend a lot of time dwelling on the pain), helplessness (feeling hopeless and helpless when in pain), and magnification (thinking the worst when in pain). A total PCS score is computed by adding all items, with high scores depicting worse coping. In the current sample, the PCS had good internal reliability (Cronbach’s *α* = 0.89).

*The Pain Anxiety Symptom Scale* (*PASS-20*) [43] is a reliable and valid measure of anxiety about pain. It has 20 items answered on a 6-point Likert scale from “never” (0) to “always” (5). Items are grouped into four subscales: avoidance (avoiding activities that cause pain), fearful thinking (fear thoughts related to pain), cognitive anxiety (difficulty thinking when in pain), and physiological response (somatic anxiety symptoms in response to pain). A total pain anxiety scale is computed by adding all items, and high scores depict worse coping. In the current sample, the PASS-20 had good internal reliability (Cronbach’s *α* = 0.88).

*The PTSD Checklist-Civilian Version* [44] is a reliable and valid 17-item measure of symptoms of post-traumatic stress disorder (PTSD). The measure provides a total severity score [45] as well as diagnostic scores. In the current sample, this questionnaire had excellent internal reliability (Cronbach’s *α* = 0.95). High scores depict worse symptoms.

*The Center for Epidemiologic Study of Depression* (*CES-D*) [46] is a widely-used, 20-item measure of self-reported depression symptoms, each answered on a 4-point Likert scale from “rarely or none of the time (less than 1 day)” (0) to “most or all of the time (5–7 days)” (3), with high scores depicting higher depression. In the current sample, the CES-D had acceptable internal reliability (Cronbach’s *α* = 0.77).

Participants completed the same battery of questionnaire with the exception of demographics at post-test and 3-month follow-up. They were emailed a link to the questionnaire via REDCap [23], a secure, electronic program used widely in research at our institution. Each participant was emailed three times and then received a call from the principal investigator prior to being considered lost to follow-up.

*Patient satisfaction.* At post-test, participants in TOR completed three questions regarding their satisfaction with the program. We used measures of patient satisfaction that evaluated the overall current satisfaction with one’s physical situation, evaluation of care delivered, and satisfaction with the personal manner of the clinician.

There were no changes to assessment or measurement after the trial commenced.

### Feasibility assessments

*Feasibility of recruitment* was assessed by determining the number of patients approached who agreed to participate.

*Acceptability of randomization and procedures* was determined by measuring loss to follow-up (post-test and 3-month follow-up) in both TOR and UC.

*Acceptability of treatment* was determined based on the number of sessions attended by participants in TOR, as well as by self-reported adverse events. At the end of the last session, the first 10 participants provided semi-structured feedback on the intervention (10–15 min interview). Patients were asked the following questions: (1) What did you think of the program? (2) If you could go back, would you choose to participate again? (3) What were the most helpful parts of the program (open ended)? and (4) Did you experience any technical difficulties? Participants also answered questions about the usefulness of each of the skills taught.

*Adherence to homework* was determined by the number of logs turned in by participants over the course of the study.

*Therapist adherence* to sessions was determined by completion of an adherence checklist by each study therapist.

*Feasibility of quantitative measures* was deemed acceptable if no questionnaires were missing in full in more than 25% of the participants and if reliability was higher than 0.70.

### Randomization and allocation concealment

Participants were randomized 1:1 to TOR or UC using a computerized random number generator program (randomnumber.org) overseen by the statistician. Only the research assistant involved in recruitment had access to the randomization sequence aside from the statistician but was not aware of the randomization status at the time of patient recruitment. Surgeons who referred participants as well as the PI were blind to intervention and control. Because we compared TOR with UC, neither the patient nor the therapist was blinded. Review of data and derivation of outcome scores was performed blind to the treatment assessment. We stopped recruitment when we achieved 50 participants with completed post-tests.

### Identification of study limitation to inform the future trial

We monitored issues related to recruitment, retention, assessments, and intervention delivery throughout the study in order to identify and rectify any emerging shortcomings that were not previously considered and testing new strategies to improve our overall methodology. The study PI maintained a constant dialog with the research assistants who collected data, the study therapists, orthopedic surgeons and patients, and the study was discussed and feedback was generated in weekly team meetings. We also monitored any adverse events in the form of self-reported increase in pain in the intervention group, none of which were observed.

### Data analyses

Feasibility studies are not designed to detect a treatment effect [21, 22]. It is recommended that feasibility trials should report primarily descriptive statistics on variables. The trial was designed to inform a multi-site hybrid efficacy-effectiveness trial. We present information on feasibility of recruitment, acceptability of screening and randomization, and feasibility of quantitative assessments. For TOR, we report on satisfaction with the program, adherence to homework, therapist adherence to treatment, and acceptability of treatment. We also present means and standard deviations (*M*, SD) for the quantitative outcomes at each time point, as well as within-subjects Cohen’s d effect sizes for improvement in participants randomized to TOR. There were no interim analyses or stopping rules for this feasibility study. Qualitative data from the open-ended questions was coded and summarized, but we did not use qualitative statistical packages to analyze it given its homogeneity.

## Results

### Sample

We present patient characteristics at baseline in Table 1, separately for TOR and UC. Patients were in majority white, educated, and women. Participants were comparable in terms of age, racial and ethnic distribution, marital and work status, and other demographic variables. However, the control group was primarily represented by women, while the gender distribution in the intervention group was balanced. There were no differences in any of the baseline characteristics between follow-up status (trial completers versus lost to follow up) (*p* > .05).

### Feasibility of recruitment

**Fig. 1:**
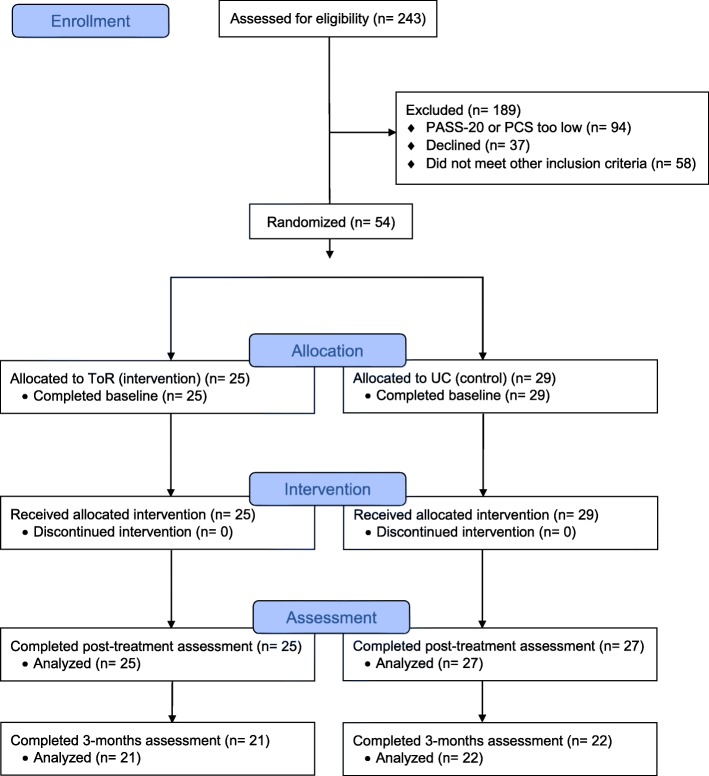
CONSORT flow diagram

A total of 243 participants were screened for eligibility; 37 declined to participate in the study (no time, no interest, did not believe they would benefit); 94 were excluded because they screened out based on PCS and PASS scores; and 58 did not meet other inclusion criteria (35 did not have a webcam or computer, 5 were on a stretcher and needed to be moved, 5 did not speak English, 2 had dementia, 1 was an inmate, and 10 agreed but changed their minds during screening). Figure 1 describes the CONSORT diagram for the study. Recruitment was most effective when surgeons introduced the study to potential patients, while cold approaching patients in the waiting room was least effective. Two of the four surgeons in the practice refused to participate in the study. One of them expressed lack of belief in psychosocial care for orthopedic trauma patients while physical recovery is in progress, while the other expressed concern about interruption in the patient flow.

### Acceptability of randomization and procedures

The CONSORT diagram shows the flow of participants in the study. Acceptability of randomization and procedures was high, and no participants dropped out after they learned their randomization status.

### Acceptability of treatment

Acceptability of treatment was high. Only one patient randomized to TOR dropped out, but this participant completed the post-test. All four sessions were scheduled on the same day and at the same time for the duration of the 4-week program. When scheduling conflicts arose, rescheduling occurred within the same week. The first ten participants in TOR who provided qualitative feedback on the program unanimously reported that they enjoyed the program and were glad they decided to participate. They noted that the program was very helpful in aiding their return to activities of daily living and putting them at ease with their recovery path. They shared that one of the toughest parts of recovery is the interruption of routine and the unknowns of the recovery process. Of the skills thought, the most useful were breath awareness, acceptance, working through myths about pain, and activity pacing. Participants loved the flexibility of the program and the live video delivery method. Neither the patients nor the therapist reported any challenges with the live video delivery of the program (e.g., internet connection, etc.), and this was regardless to whether participants used a computer, tablet, or phone.

### Adherence to homework

Participants were asked to return a home practice form weekly to the study clinician. Out of the 25 participants in TOR, 15 returned logs and practiced at least one skill. Six participants reported that they practiced skills but did not record their practice.

### Satisfaction

Satisfaction with care, clinician, and the recovery process was high (see Table 2).

**Table 2:** Baseline patient characteristics

	Intervention (TOR) (*N* = 25)		Control (UC) (*N* = 29)	
	Mean	SD	Mean	SD
Age	51	16	50	21
	*N*	%	*N*	%
Gender—women	12	48	20	69
Marital status				
Single	7	28	9	31
Married/civil union	12	48	17	58.6
Divorced/separates	3	12	2	6.9
Widowed	1	4	1	3.4
Other	2	8	0	0
Work status				
Unemployed	3	12	5	17.2
Retired	6	24	6	29.6
Homemaker	1	4	1	3.4
Employed full-time	13	52	13	44.8
Employed part-time	1	4	2	6.8
Other	1	4	2	6.8
Work-related injury—no	25	100	27	93.1
Prior orthopedic injury—yes	12	48	15	48.3
Pain medications—no	15	60	18	62.1
Anxiety/depression meds—no	18	72	18	62.1
Psychotherapy—no	23	92	23	79.3
PTSD diagnoses lifetime—no	17	68	16	55.2
Ethnicity—not Hispanic	25	100	28	96.6
Race				
White	21	84	27	93.1
Black	2	8	1	3.4
Asian	2	8	1	3.4

### Therapist adherence

Therapist’s adherence to sessions was determined by completion of adherence checklist by each study therapist. Adherence was high and therapists reported that it was easy to cover each session’s materials within the 45-min allotted time.

### Feasibility of quantitative measures

Feasibility of quantitative measures was high. No questionnaires were missing in full. Reliability was good to excellent in all questionnaires except the CES-D where reliability was acceptable (Cronbach *α* = 0.77). Participants also noted that the questionnaires were relevant and easy to understand.

### Means, standard deviations, and ranges

Means, standard deviations, and ranges for all study outcomes at all time points are depicted in Table 2.

Within-subject effect size for improvement from baseline to post-test in TOR was large for all variables (*d* = 0.83 to 2.7). The largest effect sizes were observed for SMFA (*d* = 2.7), PCS (*d* = 2.1), and PASS (*d* = 2.0), followed by pain with activity (*d* = 1.6) and pain at rest (*d* = 1.2), depression (*d* = 1.2), and PTSD (*d* = 0.8). Effect sizes for improvement in TOR between post-test and 3 months were small for SMFA (*d* = 0.23), PCS (*d* = 0.1), PASS (*d* = 0.07), pain at rest (*d* = 0.3), and depression (*d* = 0.2), and medium for pain with activity (*d* = 0.5) and PTSD (*d* = 0.7) (Table 3).

**Table 3:** Unadjusted means, standard deviations, and ranges for the outcome variables

Outcome	Baseline *M* (SD) (range)	Post-intervention *M* (SD) (range)	3-months follow-up *M* (SD) (range)
Physical function (SMFA)			
TOR	69.8 (18.2) (71.4)	20.7 (17.4) (41.2)	11.5 (19.3) (43.2)
UC	63.2 (17.4) (69.22)	48.6 (21.8) (65.2)	37.6 (30.4.0) (85)
Pain intensity at rest (NRS)			
TOR	4.4 (2.8) (9)	1.2 (1.1) (4)	0.6 (2.2) (10)
UC	1.9 (2.0) (8)	2.0 (1.9) (6)	2.1 (2.3) (9)
Pain intensity with activity (NRS)			
TOR	6.8 (2.5) (10)	2.9 (2.3) (8)	1.7 (2.2) (9)
UC	5.6 (2.6) (7)	5.1 (2.8) (9)	4.8 (3.2) (10)
Depression (CES-D)			
TOR	20.0 (12.1) (43)	8.0 (6.7) (28)	6.6 (4.8) (18)
UC	18.3 (10.9) (39)	16.7 (11.1) (40)	17.7 (12.7) (46)
PTSD (PCL-C)			
TOR	20.4 (15.2) (52)	9.2 (11.5) (33)	2.3 (4.3) (17)
UC	18.8 (13.2) (58)	17.0 (12.0) (37)	18.6 (18.7) (65)
Pain catastrophizing (PCS)			
TOR	19.7 (9.0) (35)	4.0 (5.4) (19)	4.8 (9.1) (34)
UC	17.6 (10.0)	15.1 (9.4) (24)	18.6 (13.9) (30)
Pain anxiety (PASS-20)			
TOR	41.4 (17.2) (77)	11.2 (11.7) (43)	10.1 (16.5) (57)
UC	41.8 (16.0) (72))	34.2 (19.5) (64)	39.7 (24.4) (82)
Satisfaction with recovery (0–10)		7.9 (2.2)	
Satisfaction with treatment (1–5)		4.6 (0.7)	
Satisfaction with clinician (1–5)		4.9 (0.2)	

## Discussion

We conducted a randomized controlled feasibility trial aimed at informing the design and conduct of a future multi-site phase III clinical trial with the goal of preventing chronic pain and disability in at-risk patients with orthopedic traumatic musculoskeletal injuries. While recruitment was lengthy and generally challenging, we were able to recruit the desired number of patients. Recruitment was most successful when the surgeons mentioned the study to participants. The randomization methods used in the current study successfully distributed patients equally into the TOR and UC groups; however, there was a chance difference between the groups in terms of the ratio of female to male patients. As such, stratified randomization may be employed in the phase III trial to ensure comparable gender distributions between treatment and control groups.

Once recruited and randomized, patients attended all study sessions and in majority completed post-test and follow-up questionnaires. Attrition was better than in other clinical research protocols in patients with pain [47]. However, the majority of participants were white and non-Hispanic though consistent with the typical racial and ethnic representation of the patient population at our clinic. Recruitment of a more heterogeneous ethnic sample from more ethnically diverse regions is warranted in future research. The research methodology and questionnaires were acceptable to study participants. Satisfaction with the TOR was high. There were no technical issues associated with the delivery of TOR via secure live video. Recording of homework was another area that was identified for potential improvement as 6 out of 25 patients in TOR reported taking part in homework activities but failed to record those activities in their written logs, and 4 made no report of home practice. For the phase III trial, we will implement electronic capture methods combined with text-based reminders. Due to funding restrictions, we were understaffed for the current trial and staff involved in recruitment also performed randomization. In the future multi-site feasibility trial, we will ensure that all staff but the clinician who provides the TOR intervention remain blind to allocation for the duration of the study. We did not find any differences in baseline characteristics between completers and non-completers within our sample size. In the hybrid efficacy-effectiveness definitive trial, we plan to utilize shared-baseline models for analysis to account for chance difference in baseline and incorporate all randomized participants without regard to completion of follow-up and thus would provide some protection against informative missingness.

Exploration of within-TOR effect sizes showed robust improvement in all study variables between baseline and post-test, with slight continued improvement after the end of the program and until the 3-month follow-up assessment. The largest effect sizes were for our treatment targets and for SMFA physical function, which is our primary outcome in the subsequent phase III trial. Although the small sample size prevents us for making any conclusions about the efficacy of the TOR, results provide encouraging evidence that the TOR has the potential to help improve functioning, coping, pain, and mood in patients with acute orthopedic injuries. The most robust improvement was observed for physical function, which is the proposed primary outcome in our future multisite hybrid efficacy-effectiveness design.

This feasibility trial allowed us to identify the impact of research on the normal operations of a busy orthopedic level I trauma center. We learned about the importance of integrating the research assistants within the practice and working with each individual surgeon to fit our recruitment methodology around their preferences. Two surgeons refused to allow us to recruit their patients, and one suggested that psychological factors are not important in recovery and shared his own skepticism for this work. Two surgeons were highly enthusiastic, but each required that the research assistant follows different strategies for recruitment (e.g., one surgeon wanted the research assistant to cold approach the participants in the waiting room prior to the medical visit but leave the room immediately when she was free to see the participant, and then continue recruitment after, while another surgeon introduced the study to participants and allowed the research assistant to finish recruitment before meeting with the patient). This information is important for the future study, but also for implementation purposes. The success of the multi-site hybrid efficacy-effectiveness trial will depend on securing buy-in from surgeons including providing referrals to the study. It is very likely that other trauma centers smaller than ours have a much higher patient burden which may require flexibility with study procedures. It will be important to further understand, via focus groups, barriers for participating in the recruitment process and develop educational materials to facilitate buy-in on the importance of psychosocial care for at-risk orthopedic injury patients, as well as referrals. It will be pivotal for the success of the trial to work with the surgical team and determine the best workflow for participants that will lead to highest feasibility ratings while not being disturbing of the patient flow in busy orthopedic practices. Surgeons and medical professionals in the orthopedic setting will need to be active participants in the trial design.

In conclusion, this feasibility randomized controlled trial provided rich information for the design of future research of the TOR. Lessons learned will be used to conduct a multi-site feasibility trial that include two additional geographically and ethnically diverse sites of smaller size from across the USA. Investment in a multi-site feasibility trial is mandatory in order to ensure high feasibility of the intervention and study procedures at all sites before a definitive multi-site phase III clinical trial. In doing so, researchers and clinicians can avoid wasting time and resources on running definitive trials before feasibility markers for both research and the intervention are established at all sites.

## Conclusions

We conducted a feasibility trial of the first skills-based intervention for patients with orthopedic injuries, the Toolkit for Optimal Recovery (TOR) versus usual care. We found promising evidence for feasibility and acceptability of recruitment and study procedures. Information from this feasibility trial will be used to conduct a multi-site feasibility trial at three geographically diverse trauma clinics from the USA, to set the stage for a successful phase III multi-site hybrid efficacy-effectiveness clinical trial.

## Abbreviations

CES-D: The Center for Epidemiologic Study of Depression
NRS: Numerical rating scale
NSAIDs: Nonsteroidal anti-inflammatory drugs
PASS: Pain Anxiety Symptom Scale
PCS: Pain Catastrophizing Scale
PI: Principal investigator
PTSD: Post-traumatic stress disorder
RCT: Randomized controlled trial
REDCap: Research Electronic Data Capture
SMFA: The Short Musculoskeletal Function Assessment Questionnaire
TOR: Toolkit for Optimal Recovery
UC: Usual care

## References

[1] Morris S, Lenihan B, Duddy L, O’Sullivan M (2000). Outcome after musculoskeletal trauma treated in a regional hospital. J Trauma.

[2] Macfarlane GJ (2016). The epidemiology of chronic pain. Pain..

[3] Proctor TJ, Mayer TG, Gatchel RJ, McGeary DD (2004). Unremitting health-care-utilization outcomes of tertiary rehabilitation of patients with chronic musculoskeletal disorders. J Bone Joint Surg Am.

[4] Baldwin ML (2004). Reducing the costs of work-related musculoskeletal disorders: targeting strategies to chronic disability cases. J Electromyogr Kinesiol.

[5] Vranceanu A-M, Bachoura A, Weening A, Vrahas M, Smith RM, Ring D (2014). Psychological factors predict disability and pain intensity after skeletal trauma. J Bone Joint Surg Am.

[6] Helmerhorst GTT, Vranceanu A-M, Vrahas M, Smith M, Ring D (2014). Risk factors for continued opioid use one to two months after surgery for musculoskeletal trauma. J Bone Joint Surg Am.

[7] Ring D, Kadzielski J, Malhotra L, Lee S-GP, Jupiter JB (2005). Psychological factors associated with idiopathic arm pain. J Bone Joint Surg Am.

[8] Brown GK (1990). A causal analysis of chronic pain and depression. J Abnorm Psychol.

[9] Vranceanu A-M, Safren S, Zhao M, Cowan J, Ring D (2008). Disability and psychologic distress in patients with nonspecific and specific arm pain. Clin Orthop.

[10] Vranceanu A-M, Barsky A, Ring D (2009). Psychosocial aspects of disabling musculoskeletal pain. J Bone Joint Surg Am.

[11] Crichlow RJ, Andres PL, Morrison SM, Haley SM, Vrahas MS (2006). Depression in orthopaedic trauma patients. Prevalence and severity. J Bone Joint Surg Am.

[12] Webster BS, Verma SK, Gatchel RJ (2007). Relationship between early opioid prescribing for acute occupational low back pain and disability duration, medical costs, subsequent surgery and late opioid use. Spine.

[13] Doornberg JN, Ring D, Fabian LM, Malhotra L, Zurakowski D, Jupiter JB (2005). Pain dominates measurements of elbow function and health status. J Bone Joint Surg Am.

[14] Pray JE (1991). Responding to psychosocial needs: physician perceptions of their referral practices for hospitalized patients. Health Soc Work.

[15] Archer KR, MacKenzie EJ, Bosse MJ, Pollak AN, Riley LH (2009). Factors associated with surgeon referral for physical therapy in patients with traumatic lower-extremity injury: results of a national survey of orthopedic trauma surgeons. Phys Ther.

[16] Jayakumar P, Overbeek CL, Lamb S, William M, Funes C, Gwilym S, et al. What factors are associated with disability after upper extremity injuries? A systematic review. Clin Orthop. 2018; in press. 10.1097/CORR.0000000000000427 PMC6259989 30188344

[17] Zale EL, Ring D, Vranceanu A-M (2018). The future of orthopaedic care: promoting psychosocial resiliency in orthopaedic surgical practices. J Bone Joint Surg Am.

[18] Vranceanu A-M, Beks RB, Guitton TG, Janssen SJ, Ring D (2017). How do orthopaedic surgeons address psychological aspects of illness?. Arch Bone Jt Surg.

[19] Wegener ST, Carroll EA, Gary JL, McKinley TO, OʼToole RV, Sietsema DL (2017). Trauma collaborative care intervention: effect on surgeon confidence in managing psychosocial complications after orthopaedic trauma. J Orthop Trauma.

[20] Farooq S, Akhter J, Anwar E, Hussain I, Khan SA, Jadoon I-u H (2005). The attitude and perception of hospital doctors about the management of psychiatric disorders. J Coll Physicians Surg--Pak JCPSP.

[21] Carroll KM, Rounsaville BJ (2003). Bridging the gap: a hybrid model to link efficacy and effectiveness research in substance abuse treatment. Psychiatr Serv Wash DC.

[22] Singal AG, Higgins PDR, Waljee AK (2014). A primer on effectiveness and efficacy trials. Clin Transl Gastroenterol.

[23] Leon AC, Davis LL, Kraemer HC (2011). The role and interpretation of pilot studies in clinical research. J Psychiatr Res.

[24] Lancaster GA, Dodd S, Williamson PR (2004). Design and analysis of pilot studies: recommendations for good practice. J Eval Clin Pract.

[25] Harris PA, Taylor R, Thielke R, Payne J, Gonzalez N, Conde JG (2009). Research electronic data capture (REDCap)—a metadata-driven methodology and workflow process for providing translational research informatics support. J Biomed Inform.

[26] Vlaeyen JWS, Linton SJ (2012). Fear-avoidance model of chronic musculoskeletal pain: 12 years on. Pain.

[27] Fischerauer SF, Talaei-Khoei M, Bexkens R, Ring DC, Oh LS, Vranceanu A-M (2018). What is the relationship of fear avoidance to physical function and pain intensity in injured athletes?. Clin Orthop.

[28] Stage Model for Behavioral Intervention Development [Internet]. National Institute on Aging. Available from: https://www.nia.nih.gov/research/dbsr/stage-model-behavioral-intervention-development. [cited 2018 Dec 21]

[29] Czajkowski SM, Powell LH, Adler N, Naar-King S, Reynolds KD, Hunter CM (2015). From ideas to efficacy: the ORBIT model for developing behavioral treatments for chronic diseases. Health Psychol.

[30] Vranceanu A-M, Hageman M, Strooker J, ter Meulen D, Vrahas M, Ring D (2015). A preliminary RCT of a mind body skills based intervention addressing mood and coping strategies in patients with acute orthopaedic trauma. Injury.

[31] Morone NE, Rollman BL, Moore CG, Li Q, Weiner DK (2009). A mind–body program for older adults with chronic low back pain: results of a pilot study. Pain Med.

[32] Koszycki D, Benger M, Shlik J, Bradwejn J. Randomized trial of a meditation-based stress reduction program and cognitive behavior therapy in generalized social anxiety disorder. Behav Res Ther. 2007;1(10):2518. 10.1016/j.brat.2007.04.011 17572382

[33] Ma SH, Teasdale JD (2004). Mindfulness-based cognitive therapy for depression: replication and exploration of differential relapse prevention effects. J Consult Clin Psychol.

[34] Swiontkowski MF, Engelberg R, Martin DP, Agel J (1999). Short musculoskeletal function assessment questionnaire: validity, reliability, and responsiveness. JBJS.

[35] Linton SJ (2000). A review of psychological risk factors in back and neck pain. Spine.

[36] Williams N (2016). The short musculoskeletal function assessment (SMFA) questionnaire. Occup Med.

[37] Bijur PE, Latimer CT, Gallagher EJ (2003). Validation of a verbally administered numerical rating scale of acute pain for use in the emergency department. Acad Emerg Med.

[38] Breivik H, Borchgrevink PC, Allen SM, Rosseland LA, Romundstad L, Hals B (2008). Assessment of pain. BJA Br J Anaesth.

[39] Farrar JT, Young JP, LaMoreaux L, Werth JL, Poole RM (2001). Clinical importance of changes in chronic pain intensity measured on an 11-point numerical pain rating scale. Pain.

[40] Ferreira-Valente MA, Pais-Ribeiro JL, Jensen MP (2011). Validity of four pain intensity rating scales. PAIN®.

[41] Sullivan MJL, Bishop S, Pivik J (1995). The pain catastrophizing scale: development and validation. Psychol Assess.

[42] Osman A, Barrios FX, Gutierrez PM, Kopper BA, Merrifield T, Grittmann L (2000). The pain catastrophizing scale: further psychometric evaluation with adult samples. J Behav Med.

[43] McCracken LM, Dhingra L. A short version of the Pain Anxiety Symptoms Scale (PASS-20): preliminary development and validity [Internet]. Pain Research and Management. 2002. Available from: https://www.hindawi.com/journals/prm/2002/517163/abs/. [cited 2018 Aug 3] 10.1155/2002/517163 16231066

[44] FW Weathers, Litz B, Herman D, Huska J, Keane T. The PTSD checklist (PCL): reliability, validity, and diagnostic utility. [Internet]. National Center for PTSD; 1993. Report No.: Paper presented at the 9th annual conference of the ISTSS. Available from: https://www.ptsd.va.gov/professional/assessment/adult-sr/ptsd-checklist.asp. [cited 2018 Aug 3]

[45] Diagnostic and statistical manual of mental health disorders. (4^th^ ed). Washington: American Psychiatric Association; 2000.

[46] Radloff LS (1977). The CES-D scale: a self-report depression scale for research in the general population. Appl Psychol Meas.

[47] Veehof MM, Oskam M-J, Schreurs KMG, Bohlmeijer ET (2011). Acceptance-based interventions for the treatment of chronic pain: a systematic review and meta-analysis. PAIN®.

